# NEIGHBORHOOD POLLUTING INFRASTRUCTURE AND COGNITIVE HEALTH IN OLDER ADULTS

**DOI:** 10.1093/geroni/igae098.3215

**Published:** 2024-12-31

**Authors:** Vivian Ku, Ketlyne Sol, Laura Zahodne

**Affiliations:** University of Michigan, Ann Arbor, Michigan, United States; University of Michigan, Ann Arbor, Michigan, United States; University of Michigan, Ann Arbor, Michigan, United States

## Abstract

Air pollution is known to be associated with poorer brain and cognitive health. Modifiable sources of pollution in neighborhoods such as industrial and hazardous waste facilities may serve as intervention targets. However, less is known about how polluting infrastructure relates to different cognitive domains. This study examines the relationship between polluting infrastructure and cognition within older adults. 475 adults aged 55+ from the Michigan Cognitive Aging Project (MCAP) completed a comprehensive neuropsychological battery measuring cognition across five domains (episodic memory, executive function, processing speed, language, visuospatial function). Participant geocoded addresses were linked to data on polluting infrastructure in the National Neighborhood Data Archive. Polluting infrastructure was operationalized as the presence of Toxic Release Inventory sites and the proportion of highway length to the total length of streets in a census tract. Generalized estimating equations accounting for geographic clustering modeled relationships between polluting infrastructure and cognitive health, controlling for age, sex, education, income, population density, and neighborhood disadvantage. Higher proportions of highways were related to poorer episodic memory (β= -0.660, p= 0.040). A trend was also found between the presence of at least one toxic site and poorer language (β= -0.121, p= 0.056). Both highways and toxic sites represent polluting infrastructure that could reduce cognitive health among older adults. Differential associations between pollution sources and cognitive domains may indicate that different types of pollutants (e.g., airborne fine particulates versus others) influence unique brain pathways.

